# Supplementary material for: Tff3 Deficiency Differentially Affects the Morphology of Male and Female Intestines in a Long-Term High-Fat-Diet-Fed Mouse Model
Source: Int J Mol Sci. 2023 Nov 15;24(22):16342. doi: 10.3390/ijms242216342 (PMC10671422; doi:10.3390/ijms242216342)
Supplement: Supplementary file 1 [file ijms-24-16342-s001.zip › ijms-2605601-supplementary.pdf]

International Journal of
Molecular Sciences

MDPI

Supplementary Materials:

Supplementary Figures:

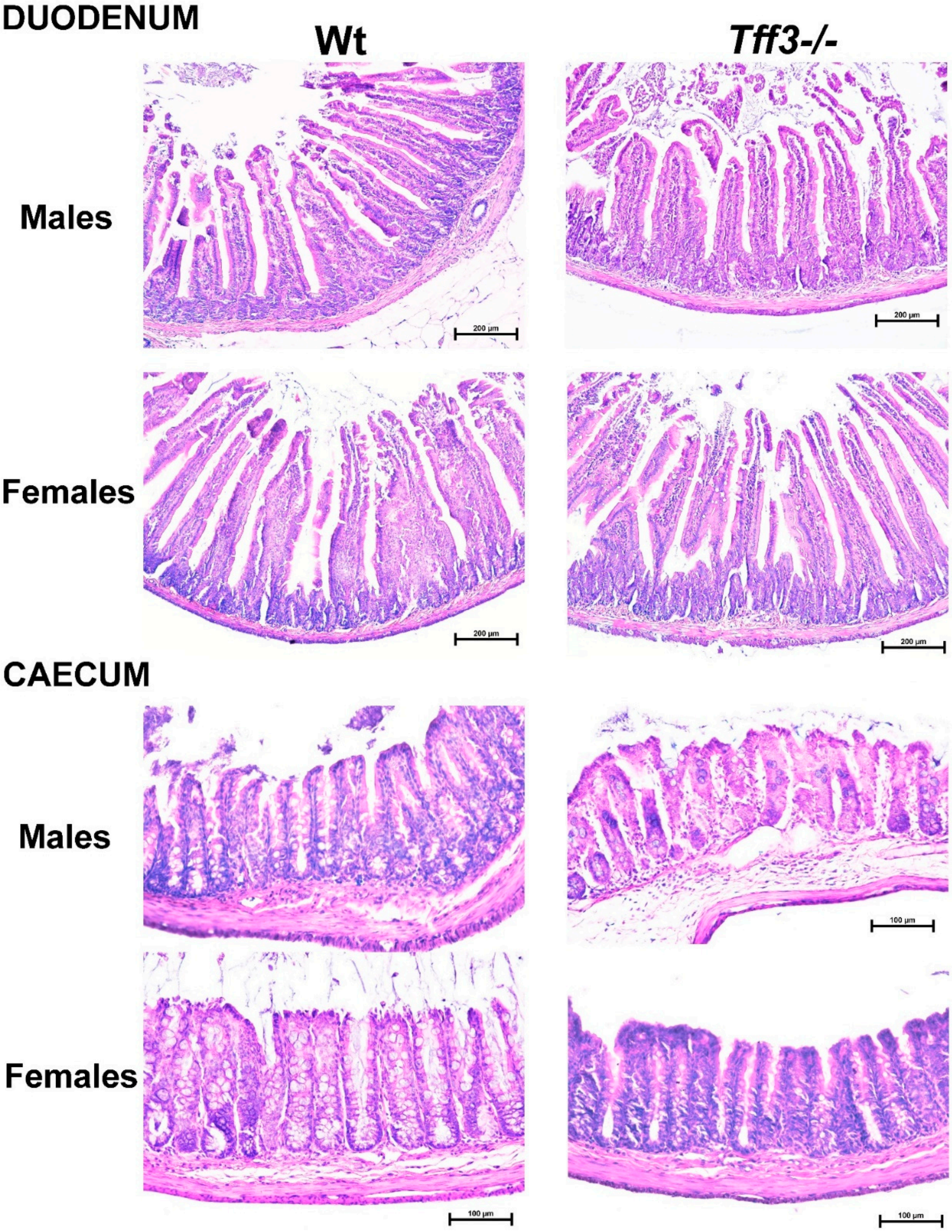

Figure S1. Representative photomicrographs of the duodenum and cecum of Wt and *Tff3* -/- mice. H&E staining; scale bars 200 µm (duodenum) and 100 µm (cecum).

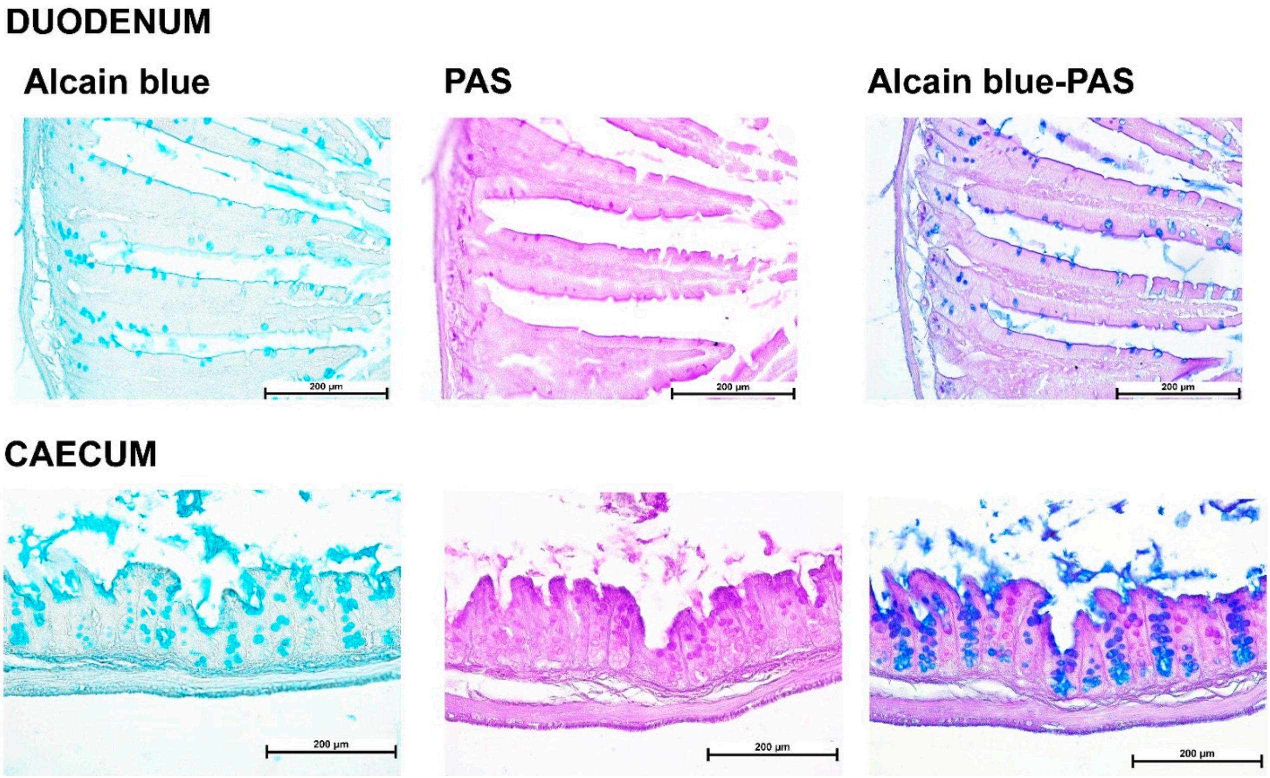

Figure S2. Representative photomicrographs of the duodenum and cecum stained with Alcain Blue/PAS. Representative histological sections of the duodenum and cecum were stained with Alcian blue solution (pH 2.5) and PAS, separately or as a combined Alcian blue–PAS staining. Acidic and neutral mucins in goblet cells were stained with Alcian blue and PAS pale blue and magenta, respectively. Goblet cells containing a mixture of neutral and acidic mucins were stained blue–purple by combined Alcian blue–PqAS staining (right column panels). Scale bar, 200 μm.

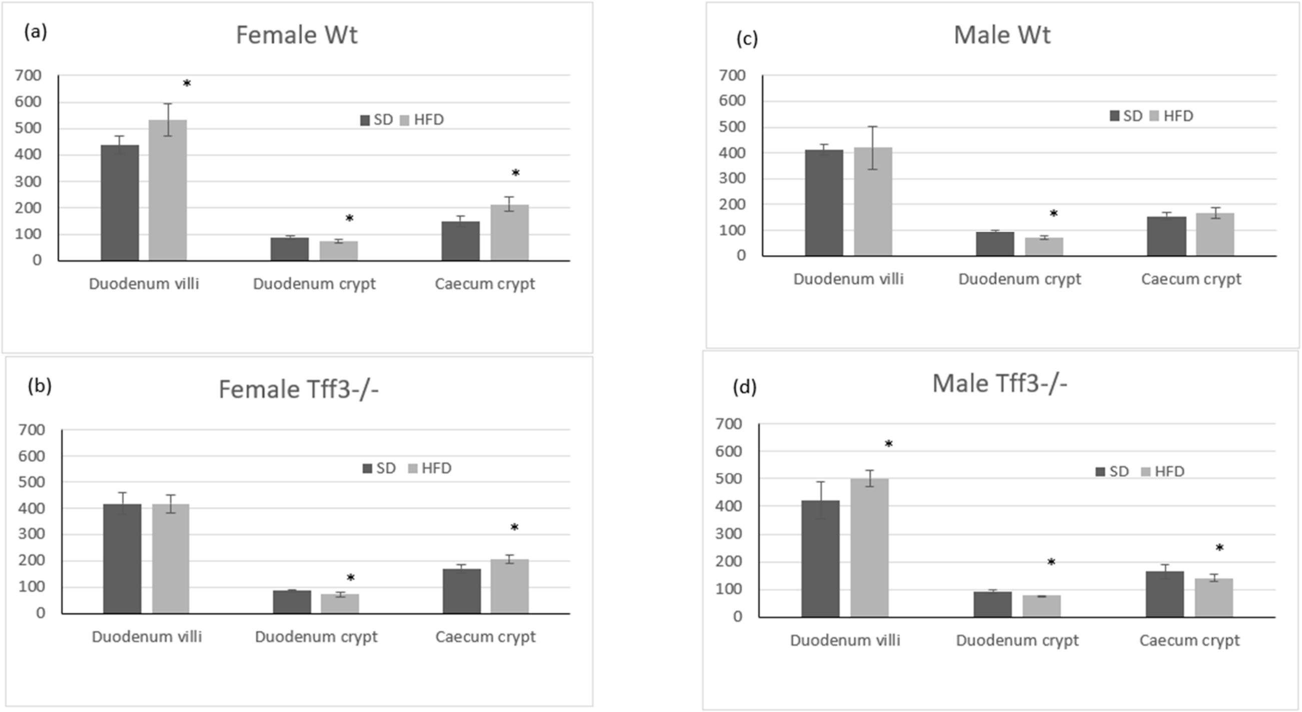

Figure S3: Average height of villi, depth of crypts in the duodenum, and crypt depth in the cecum. On the abscissa are different parts of the intestine: the duodenum and cecum on the ordinate are the height in μm, SD—standard diet (5 animals per group), HFD—high fat diet (10 animals per group).), (a) female WT, (b) female Tff3-/-, (c) male WT, (d) male Tff3-/-. * significan difference among SD and HFD ($p \leq 0.05$)

Supplementary tables:

Table S1. Chemical analysis (g/kg diet) of HFD and SD groups.

	**High fat diet (HFD)**	**Standard diet (SD)**
Dry matter (g/kg)	926.5	917.1
Crude fiber (g/kg)	42.3	60.1
Crude fats (g/kg)	324.9	26.4
Crude protein (g/kg)	237.3	202.4
Crude ash (g/kg)	56.1	62.8
Non-nitrogen extractives (g/kg)	265.9	565.4
Minerals:		
Na (g/kg)	2.4	2.5
K (g(kg)	10.9	11.8
Ca (g/kg)	8.4	8.3
Mg (g/kg)	2.2	2.2
P (g/kg)	8.2	7.1

Table S2. Oligonucleotides used for qPCR analysis

Gene Symbol	Accession No.	Primer Sequence Forward (5′-3′) Reverse (5′-3′)	Optimized qPCR Conditions (Annealing Temp/MgCl)
		ERS markers	
ATF4	NM_009716.3	CCACTCCAGAGCATTCCTTTAG<br>CTCCTTTACACATGGAGGGATTAG	59 °C; 3.5 mM
BIP	NM_001163434.1	GAGACTGCTGAGGCGTATTT<br>CAGCATCTTTGGTTGCTTGTC	58 °C; 3.5 mM
CHOP	NM_007837.4	TTGAGCCTAACACGTCGATTAT<br>CACTTCCTTCTGGAACACTCTC	58 °C; 3 mM
EDEM	NM_138677.2	TGAAAGCATGTGAGGGTAGTG<br>GAGAGAAGGGAAGACAGGATAGA	61 °C; 3.5 mM
GRP94	NM_011631.1	AAGAATGAAGGAAAAACAGGACAAAA<br>CAAATGGAGAAGATTCCGCC	58 °C; 3 mM
sXBP1	NM_008934.4	GAGTCCGCAGCAGGTG<br>GTGTCAGAGTCCATGGGA	56 °C; 3 mM
usXBP1	NM_008934.4	CAGCACTCAGACTATGTGCA<br>GTCCATGGGAAGATGTTCTGG	59°C; 3,5mM
		Cytokines	
CCR2	NM_009915.2	GGTCTGGTTGGGTTGTAAA<br>GTCTTTGAGGCTTGTTGCTATG	59 °C; 3 mM
CD68	NM_001291058.1	CTCTTGCTGCCTCTCATCATT<br>CTGGTAGGTTGATTGTCGTCTG	58 °C; 2,5 mM
CXCL1	NM_008176.3	GTGTCAACCACTGTGCTAGT<br>CACACATGTCCTCACCCTAATAC	61 °C; 3.5 mM
CXCR7	NM_001271607.1	GACCATGTAGGCCTCAGATTAG<br>CAGCCGAGACTGGCATAAA	63 °C; 3.5 mM
IL1α	NM_010554.4	CCTTACACCTACCAGAGTGATTT<br>CCTTACACCTACCAGAGTGATTT	65 °C; 3 mM
IL1β	NM_008361.4	ATGGGCAACCACTTACCTATTT<br>GTTCTAGAGAGTGCTGCCTAATG	64 °C; 3 mM
IL6	NM_031168.2	GATAAGCTGGAGTCACAGAAGG<br>TTGCCGAGTAGATCTCAAAGTG	59 °C; 3.5 mM
IL-14	NM_001005506.3	CCTCACTTCAGCTACCTCTTAAA<br>CTACAAGTGGATGGAGGGAAAG	61 °C; 3.5 mM
MCP1	NM_011333.3	CCTGGATCGGAACCAAATGA<br>CGGGTCAACTTCACATTCAAAG	62 °C; 3 mM
TGFα	NM_031199.4	CTTTAGGAAGGACCTGGGTTG<br>GTGTGTCCAGGCTCCAAATA	66 °C; 3 mM
TNFα	NM_013693.3	GTCTCAGAATGAGGCTGGATAAG<br>CATTGCACCTCAGGGAAGAA	63 °C; 2.5 mM
		Oxidative stress markers	

IGF	NM_010512.5	TAACATCCGGAGAGGCAATAACAGAC-<br>CAAGGCATGAGAATGG	59 °C 3 mM
NOX2	NM_007807.5	ACTCCTTGGGTCAGCACTGG<br>GTTCCTGTCCAGTTGTCTTCG	62 °C 3 mM
SOD1	NM_011434.2	GCCTTCTGCTCGAAGTGGAT<br>GGAAGCATGGCGATGAAAGC	59 °C 3.5 mM
SOD3	NM_011435.3	TGGCTGATGGTTGTACCCTG<br>TGAGAAGATAGGCGACACGC	60 °C 2.5 mM
		Apoptosis	
AIFM1	NM_012019.3	CGGGAGGTCAAGTCAATTACAG<br>GGAACAGCTGGATCACTTCTATG	61 °C 3.5 mM
BAG6	NM_057171.3	TTTCTTCCACCAGCACTACC<br>GGCTGAACCTGTACCAAAGA	59 °C 3,5 mM
BCL2	NM_009741.5	CCCTGGACGATTCAGCATTTA<br>CTCTGCTTCCCATCTGGATTT	61 °C 3 mM
TLR4	NM_021297.3	TGGTCAGTGTGATTGTGGTATC<br>GCTTTCTCCTCTGCTGTACTT	58 °C 3 mM
		Referent (housekeeping) genes	
ACTB	NM_007393.5	GCAAGCAGGAGTACGATGAG<br>CCATGCCAATGTTGTCTCTT	61 °C; 3.5mM
B2M	NM_009735.3	CCTGCAGAGTTAAGCATGACAGT<br>TCATGATGCTTGATCACATGTCT	60 °C; 3 mM

Table S3. Histological measurements: height of the villi in the duodenum, and depth of the crypt in the duodenum and cecum (average ± standard deviation) of mice fed a standard diet (SD).

**Intestinal histology**	**Wt male (5)**	***Tff3*-/- male (5)**	**Wt female (5)**	***Tff3*-/- female (5)**
Duodenum				
Villi height (μm)	410.4 ± 18.8	419.8 ± 60.3	436.9 ± 31.2	416.9 ± 37.3
Crypt depth	93.9 ± 4.0	92.2 ± 4.6	88.6 ± 6.0	86.7 ± 4.3
Ratio villi : crypt	4.4 : 1	4.6 : 1	4.9 : 1	4.8 : 1
Cecum				
Crypt depth	154.9 ± 212.5	164.9 ± 23.6	150.1 ± 18.5	170.9 ± 13.6

Results are presented as average ± standard deviation
